# Draft Genome Sequence of the Psychrotolerant Bacterium *Methylobacterium* sp. Strain BTF04, Isolated from Freshwater in Antarctica

**DOI:** 10.1128/MRA.00171-20

**Published:** 2020-05-21

**Authors:** Ahnna Cho, Yong-Joon Cho, Soyeon Kim, Ok-Sun Kim

**Affiliations:** a Division of Polar Life Sciences, Korea Polar Research Institute, Incheon, Republic of Korea; b Department of Biological Sciences and Research Institute of Basic Sciences, Seoul National University, Seoul, Republic of Korea; Loyola University Chicago

## Abstract

*Methylobacterium* sp. strain BTF04, a pink-pigmented psychrotolerant bacterium, was isolated from freshwater on Barton Peninsula, King George Island, Antarctica. Here, we report the assembled draft genome sequence of *Methylobacterium* sp. strain BTF04.

## ANNOUNCEMENT

*Methylobacterium* sp. strain BTF04, belonging to the class *Alphaproteobacteria*, was isolated from a freshwater (wetland) sample collected on Barton Peninsula, King George Island, Antarctica (62°14′17.0″S, 58°43′46.6″W) on 25 January 2015. This wetland is produced by melting snow and ice during the Antarctic summer period (December to February). The genus *Methylobacterium* has potential for ecological contributions to the promotion of plant growth, biotechnology, and biodegradation. In particular, this group can be used to remove environmental contamination due to its ability to biodegrade toxic substances and its resistance to heavy metal (1).

Interestingly, the growth temperature of the genus *Methylobacterium* ranges from 15°C to 35°C, and the species belonging to this genus have been found in various terrestrial habitats, such as leaf surfaces and nodules, soil, dust, freshwater, Antarctic subglacial lakes, drinking water, and oil-contaminated sites (2–5). However, unlike other strains, BTF04 was cultivated at 10°C on a 0.1% R2A agar plate. This strain was closely related to the M. goesingense iEll3 type strain and the M. adhaesivum AR27 type strain at 99.36% and 99.35%, respectively, by 16S ribosomal DNA typing using universal 27F-1492R primers and the EzTaxon-e database (6). Here, we report the genome sequence of the psychrotolerant bacterium *Methylobacterium* sp. strain BTF04.

Genomic DNA was extracted from *Methylobacterium* sp. strain BTF04 using a PowerSoil DNA isolation kit (Qiagen, USA). A DNA library for next-generation sequencing (NGS) was prepared using a Nextera DNA Flex library prep kit (catalog number 20018704; Illumina, USA) following the manufacturer’s protocol. The sequencing was performed by Illumina MiSeq with 300-bp paired-end cycles. Raw data of 185,394 paired-end reads were obtained, and adapter removal and quality-trimming were performed using Trimmomatic v0.36 with default parameters (7). Assembly was performed using SPAdes v3.11.0 in multicell mode, along with read error and mismatch correction (8). As a result of the assembly, a 5,188,880-bp genome was obtained with 259 contigs, an *N*_50_ value of 103,303 bp, a GC content of 66.5%, and genome coverage of 21.44×. Gene prediction and annotation were performed using the NCBI Prokaryotic Genome Annotation Pipeline v4.11 (9), and functional annotation and KEGG pathway mapping were performed using BlastKOALA v2.2 against the “species_prokaryotes” database (10). The genome has a total of 4,921 genes, including 4,865 coding genes, 3 rRNAs, 49 tRNAs, 4 noncoding RNAs (ncRNAs), and 172 pseudogenes.

Many *Methylobacterium* species are known for their metal tolerance, and the genome of *Methylobacterium.* sp. strain BTF04 also has genes involved in heavy metal resistance, such as heavy metal translocating P-type ATPase (*G3T14_24095*), CusA/CzcA family heavy metal efflux RND transporter (*G3T14_19645*), nickel-responsive transcriptional regulator NikR (*G3T14_03080* and *G3T14_22525*), nickel transporter (*G3T14_00040* and *G3T14_10395*), cobalt transporter (*G3T14_12875*), nickel/cobalt efflux transporter RcnA (*G3T14_18340*), and cadmium-translocating P-type ATPase (*G3T14_20510*). There are three genes encoding cold shock protein (*G3T14_12600*, *G3T14_14945*, and *G3T14_16670*), which are supposed to help in adaptation to low-temperature environments, such as Antarctica. The genome information from strain BTF04, a *Methylobacterium* strain specialized in cold environments, gives insights into this microorganism’s strategy for survival in its habitat and is useful for comparative genomics of this genus, which is found everywhere on our planet.

### Data availability.

The genome sequences and annotations were deposited in GenBank under the accession number JAAHTB000000000 and can be found under BioProject accession number PRJNA605674.
